# Case series of non-ampullary duodenal adenomas

**DOI:** 10.1016/j.amsu.2021.102730

**Published:** 2021-08-20

**Authors:** Amitabh Yadav, Samiran Nundy

**Affiliations:** Dept. of Surgical Gastroenterology and Liver Transplant, Sir Ganga Ram Hospital, 110060, New Delhi, India

**Keywords:** Sporadic duodenal adenoma, Familial adenomatous polyposis, Tubulo-villous adenoma

## Abstract

Duodenal adenomas are benign tumours of the duodenum which carry a malignant potential. They are found either sporadically or associated with familial syndromes. Majority of these cases are treated endoscopically but surgical resection is a better alternate to endoscopy in select cases. Endoscopic treatment is associated with higher chances of local recurrence and require frequent check endoscopies in the follow up period, while surgery offers a one-time treatment option. Identification of the ampulla and a duodenal resection sparing ampullary area becomes difficult in larger lesions of the 2nd part of the duodenum. Passage of a catheter from cystic duct through common bile duct to duodenum aids in identification of the ampullary area and is helpful in performing a local/wedge resection of the duodenum containing adenoma without injuring ampullary orifice.

## Highlights

• Duodenal adenomas carry malignant potential.
• Found sporadically or with familial syndromes.
• Surgery offers one time treatment options for larger lesions.
• Isolating the ampullary orifice through catheterization of the cystic duct is useful.

## 1 Introduction

Duodenum is about 8% of the total length of small bowel but it is the site for 10–22% of the small bowel tumours. Adenomas are benign epithelial tumours, and the most common variety of polyps in the duodenum. They are either sporadic or may be associated with hereditary syndromes, like Familial Adenomatous Polyposis (FAP) and Peutz- Jeghers syndrome. They can be ampullary or non-ampullary according to the site of their origin. Non-ampullary duodenal adenomas are commonly found in patients of FAP, while sporadic occurrence is rare [1,2].

We operated two cases of large non-ampullary duodenal adenomas in the department of Surgical Gastroenterology and Liver Transplant at a tertiary care teaching hospital. The identification of the ampullary area was difficult in both the cases due to large size of the adenoma causing almost complete occlusion of the duodenal lumen. One case was of sporadic variety, and the other was associated with FAP. The aim of the article is to report these cases and review of literature on this subject. This case series has been reported in line with the PROCESS Guidelines [3].

### 1.1 Case 1

A 46-year-old male presented with recurrent abdominal pain and vomiting for six months with recent exacerbation of symptoms. Clinical examination was unremarkable and blood investigations revealed microcytic hypochromic anaemia with normal tumour markers.

**Fig. 1:**
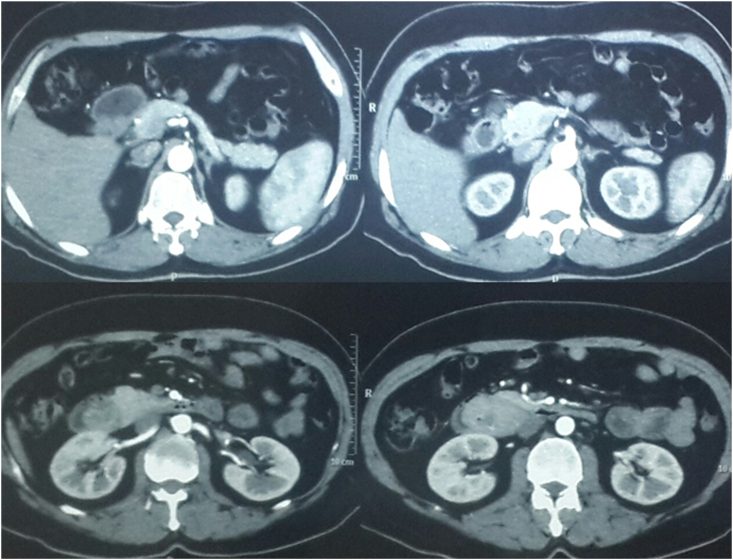
Coronal images of CECT of first case – origin of adenoma in first part of duodenum, extending and filling the second part of duodenum.

**Fig. 2:**
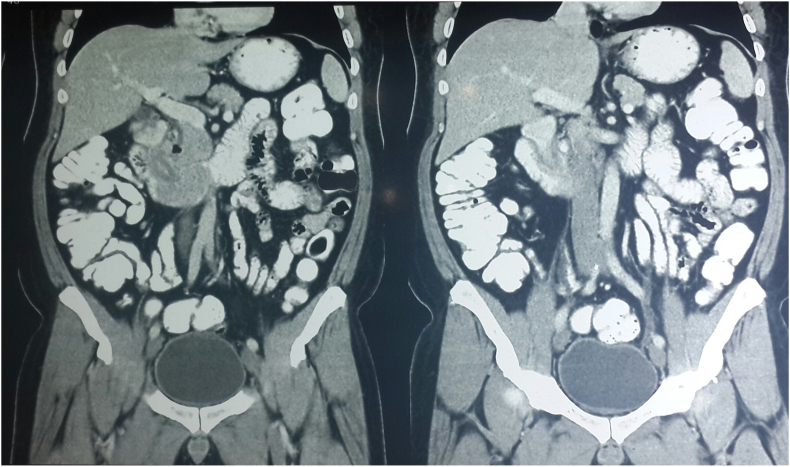
Sagittal images of CECT of first case – showing complete filling of the second part of duodenum.

**Fig. 3:**
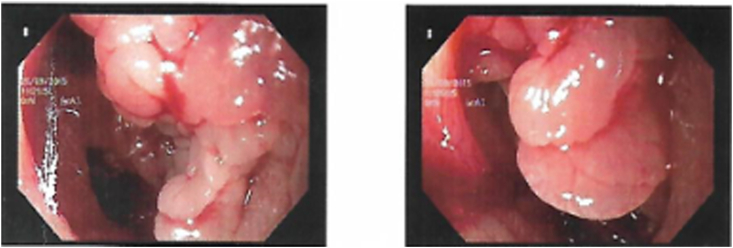
Endoscopy pictures of Duodenal Adenoma of the first case.

Contrast enhanced CT scan of the abdomen revealed a large, ill-defined heterogeneously enhancing, hypodense mass within 2nd part of the duodenum which appeared resectable (Fig. 1, Fig. 2). Upper Gastrointestinal (UGI) endoscopy revealed a large, ulcerated lesion at D1 - D2 junction projecting further into the duodenum. The ampullary area was not identified separately from the lesion (Fig. 3). The endoscopic biopsy of the lesion showed tubulo-villous adenoma. The patient was optimized and taken as an elective case for an open surgical procedure.

**Fig. 4:**
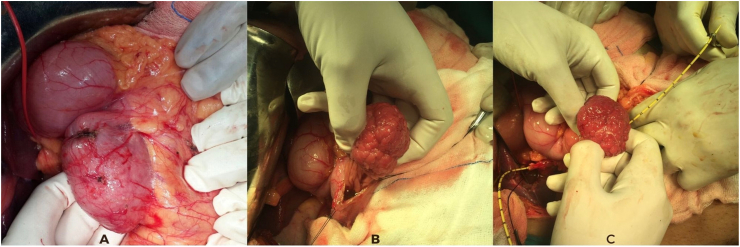
Intra-operative images A: mobilized duodenum with adenoma inside, B: vertical enterotomy made and adenoma delivered, C: cholecystectomy done, and ureteric catheter passed from cystic duct to ampulla.

Intra-operatively, a large mass (8 × 4x3 cm with a very wide stalk) arising from the mesenteric border of the junction of the first and second part of the duodenum was found, which was causing luminal obstruction. Ampullary area could not be identified separately so a small catheter was introduced from cystic duct to ampulla (after cholecystectomy) (Fig. 4) and an ampulla preserving duodenal resection with gastrojejunostomy and feeding jejunostomy was done. Frozen section showed tubulovillous adenoma. The patient had an uneventful post-operative course and was discharged on day 5 of surgery. The final biopsy showed tubular adenoma. 6 years follow up showed no long-term complications including recurrence.

### 1.2 Case 2

A 26-year-old male presented with pain abdomen and vomitings for 4–5 months. Ultrasound abdomen suggested agenesis of left kidney with transient duodenal intussusception. CT scan showed a circumferential pyloroduodenal thickening, intussuscepting into duodenum with? neoplastic mass as a lead point. UGI Endoscopy showed a polypoidal growth at D1-D2 junction causing luminal narrowing and scope negotiated beyond with mild resistance. Endosonography (EUS) showed 38 × 32 mm lesion at D1-D2 junction, arising from 3rd layer of the duodenal wall. Biopsy and EUS guided *trans*-duodenal FNAC were inflammatory only.

Intra-operatively, posterior wall of second part of the duodenum had a 6x5x1.5 cm sized polypoidal friable mass. Ampulla was not separately visualised, so the ampullary area was spared after passage of a small catheter via cystic duct to ampulla. Wedge resection of the duodenum with retro colic gastrojejunostomy and feeding jejunostomy were done. Post-operative period was uneventful, and he was discharged on 6th post-operative day. Biopsy was suggestive of adenomatous polyp with low grade dysplasia.

The patient had no complications or recurrence in the one-year follow-up till the article was submitted for publication.

## 2 Discussion

Incidence of duodenal polyps varies from 1.02% to 4.6% of all the patients referred for upper gastrointestinal endoscopy [4,5]. The sporadic polyps in the duodenum are classified depending upon their location and histopathological characteristics. Duodenal adenomas are sporadic in 40% cases, associated with FAP in 60% [4,6], and have 30–85% chances of conversion to malignancy [4,7]. Mostly they are flat or sessile lesions located on the posterior or lateral wall of the second part of duodenum [1,8]. This is a premalignant condition and the factors promoting development of carcinoma from an adenoma are number, size, location, severity of dysplasia and villous histology [9]. Lesions larger than 2 cm and ampullary location carries a higher risk of malignant change. (4) It may take up to two decades for development of adenocarcinoma from low grade dysplasia [1,10,11].

Recent evidence suggests that, regardless of their types and location, duodenal adenomas are similar in morphology and molecular characteristics to colorectal adenomas and similar mechanisms are involved in their conversion to adenocarcinoma [1]. Higher concentration of bile acids and pancreatic secretions in the duodenum is thought to be responsible for development of malignant change. Small bowel adenocarcinomas are preceded by adenomas, as seen in colonic cancers, and this is evident by finding of residual adenomatous tissue on histopathology either within or adjacent to the adenocarcinoma [12].

Genetic mutations, like those in Adenomatous Polyposis Coli (APC), p53 and KRAS, are thought to be responsible for progression of adenoma to carcinoma. CpG Island Methylator Phenotype (CIMP) is well recognized phenotype found in patients with colorectal polyps and cancers. Its role has also been suggested in small bowel cancers and a positive status is associated with larger lesions, associated dysplasia, ampullary location and villous subtype and are associated with poor prognosis. These patients require more aggressive surveillance and treatment [2].

FAP is an autosomal dominant disorder which is caused by mutant APC gene on the long arm of chromosome 5. The lifetime risk of developing duodenal malignancy in these patients of FAP is about 3–5% [13]. A staging system was developed by Spigelman for assessment of severity and risk of development of malignancy in duodenal adenomas in FAP. This was based on number of adenomas, size, histological types, and degree of dysplasia. The score based on these parameters was calculated and stages from 0-IV were given [14]. The risk is low in stages 0 to III (0.7%) and high in stage IV (7–36%) over a follow up of 7.6–10 years [4,14,15].

Histopathology plays an important role in the management of duodenal adenomas. In patients with non-ampullary solitary adenomas and low-grade dysplasia, the risk of malignant conversion is low but about 20% develop high grade dysplasia and 4.7% develop noninvasive malignancy. A close follow up is required in these patients. Patients with high grade dysplasia and a lesion larger than 2 cm have a higher risk of progression to malignancy, hence should be treated immediately [4,10].

Most lesions are amenable to endoscopic resections. The endoscopic treatments recommended are 1) Endoscopic Polypectomy (lesions <1 cm), 2) Endoscopic Mucosal Resection (EMR) for larger lesions and 3) Ablation by Argon Plasma Coagulation (APC). The risk of complications with EMR increases with the increase in size of the lesion. It is indicated for lesions less than 2 cm in size and less than 33% of duodenal circumference involvement [1].

Disadvantages of EMR are bleeding (43%), recurrence (14–37%) and perforation risk (4.3%) [[16], [17], [18], [19]]. Disadvantage with APC is non availability of entire specimen for histology, so it is mainly used as an adjunct to EMR. Endoscopic submucosal dissections (ESD) are not done in duodenum owing to thin wall and risk of perforation [20]. Follow up check endoscopy is required after 3–6 months of endoscopic treatment and then at 6 to 12 monthly intervals [21]. In larger lesions endoscopy usually never achieves a polyp free duodenum.

Surgery is a one-time treatment option and is the preferred mode of treatment for lesions which are >2 cm in size, recurrent or show severe dysplasia. Surgical procedures recomemded are trans duodenal submucosal or wedge resection, segmental duodenal resection sparing pancreas and pancreaticoduodenectomy or Endoscopy assisted laparoscopic resections [22]. Whipple's operation is preferred in cases with positive frozen section, and large or multicentric benign Villous Tumour of the Duodenum (VTD). Factors favouring malignancy include severe dysplasia, hard area on palpation, ulceration, biliary or pancreatic obstruction, and villous lesions extending into bile/pancreatic duct. In the presence of these factors, pancreatoduodenectomy is strongly recommended. In the absence of above findings, local resection is advised [7,23]. For lesions involving the distal 3rd or 4th part of duodenum, pancreas sparing duodenectomy with extended resection is a reasonable option. Literature showed that the recurrence rates of VTD after trans duodenal local excision was around 32% at 5 years and 24% of these recurrences were malignant [24].

For lesions larger in size and located near the ampullary area, difficulty arises in performing an ampulla preserving wedge/local resection. To overcome this same problem in both the above cases, we attempted a cholecystectomy and passed a small catheter from the cystic duct stump to the lower end of bile duct. It was then taken out through ampulla which helped in identifying the ampullary area. The catheter acted as a guide and an ampulla sparing resection was done. We found this technique useful, but it needs further research and data support before being recommended in clinical practice.

## 3 Conclusion

Surgery is a better and one-time option than endoscopy to treat large duodenal adenomas. Adding cholecystectomy and passage of a catheter from cystic duct across ampullary region helps in identification of the ampulla thus, avoiding its injury during local resection.

## Informed consent

Written informed consent was obtained from the patients for publication of this case report and accompanying images. A copy of the written consent is available for review by the Editor-in-Chief of this journal on request.

## External funding

There are no sources of funding **for this research.**

## Ethical approval

Not Required for case reports as per our institution policy.

## Provenance and peer review

Not commissioned, externally peer-reviewed.

## Author contribution

1. Amitabh Yadav - Corresponding author - study concept, data collection, data analysis or interpretation, writing the paper
2. Samiran Nundy - Study design and editing

## Registration of research studies

1. Name of the registry: researchregistry.com
2. Unique Identifying number or registration ID: researchregistry6837
3. Hyperlink to your specific registration (must be publicly accessible and will be checked): https://www.researchregistry.com/browse-the-registry#home/

## Guarantor

Dr Amitabh Yadav.

Consultant.

Surgical Gastroenterology and Liver Transplant.

Sir Ganga Ram Hospital.

New Delhi.

## Declaration of competing interest

All authors declares that there is no conflict of interest.

## References

[1] Aleksandra P.M., Sanja, Miodrag K., Milica S.L., Tomica (2019). Assessment of duodenal adenomas and strategies for curative therapy. Dig. Dis..

[2] Sun L., Guzzetta A.A., Tao F., Chen J., Jeschke J. (2014). CpG island methylator phenotype and its association with malignancy in sporadic duodenal adenomas. Epigenetics.

[3] Agha R., Sohrabi C., Mathew G., Franchi T., Kerwan A., al e (2020). The PROCESS 2020 guideline: updating consensus preferred reporting of CasESeries in surgery (PROCESS) guidelines. Int. J. Surg..

[4] Lim C.H., Cho Y.S. (2016). Nonampullary duodenal adenoma: current understanding of its diagnosis, pathogenesis, and clinical management. World J. Gastroenterol..

[5] Jung S.H., Chung W.C., Kim E.J., Kim S.H., Paik C.N. (2010). Evaluation of non-ampullary duodenal polyps: comparison of non-neoplastic and neoplastic lesions. World J. Gastroenterol..

[6] Culver E.L., McIntyre A.S. (2011). Sporadic duodenal polyps: classification, investigation, and management. Endoscopy.

[7] Farnell M.B., Sakorafas G.H., Sarr M.G., Rowland C.M., Triotos M. (2000). Villous tumors of the duodenum: reappraisal of local vs. extended resection. ScienceDirect.

[8] Ma M.X., Bourke M.J. (2017). Management of duodenal polyps. Best Pract. Res. Clin. Gastroenterol..

[9] Walton S., Kallenberg F., Clark S., Dekker E., Latchford A. (2016). Frequency and features of duodenal adenomas in patients with MUTYH-associated polyposis. Clin. Gastroenterol. Hepatol..

[10] Okada K., Fujisaki J., Kasuga A., Omae, Kubota M. (2011). Sporadic nonampullary duodenal adenoma in the natural history of duodenal cancer: a study of follow-up surveillance. Am. J. Gastroenterol..

[11] Vasen H.F., Möslein Alonso A., Aretz S., Bernstein I. (2008). Guidelines for the clinical management of familial adenomatous polyposis (FAP). Gut.

[12] Sellner (1990). Investigations on the significance of the adenoma-carcinoma sequence in the small bowel. Cancer.

[13] Mathus-Vliegen E.M.H., Boparai K.S., Dekker E., Nv Geloven (2011). Progression of duodenal adenomatosis in familial adenomatous polyposis: due to ageing of subjects and advances in technology. Fam. Cancer.

[14] Spigelman A.D., Williams C.B., Talbot I.C., Domizio Phillips RK. (1989). Upper gastrointestinal cancer in patients with familial adenomatous polyposis. Lancet.

[15] Bülow, Björk, Christensen I.J., Fausa O., Järvinen H. (2004). Duodenal adenomatosis in familial adenomatous polyposis. Gut.

[16] Klein A., Nayyar D., Bahin F.F., Qi Z., Lee (2016). Endoscopic mucosal resection of large and giant lateral spreading lesions of the duodenum: success, adverse events, and long-term outcomes. Gastrointest. Endosc..

[17] Nonaka S., Oda I., Tada K., Mori G., Sato Y. (2015). Clinical outcome of endoscopic resection for nonampullary duodenal tumors. Endoscopy.

[18] Navaneethan U., Lourdusamy D., Mehta D., Lourdusamy V., Venkatesh P. (2014). Endoscopic resection of large sporadic non-ampullary duodenal polyps: efficacy and long-term recurrence. Surg. Endosc..

[19] Seo J.Y., Hong S.J., Han J.P., Jang H.Y., Myung Y.S. (2014). Usefulness and safety of endoscopic treatment for nonampullary duodenal adenoma and adenocarcinoma. J. Gastroenterol. Hepatol..

[20] Pimentel-Nunes P., Dinis-Ribeiro M., Ponchon T., Repici A., Vieth M. (2015). Endoscopic submucosal dissection: European society of gastrointestinal endoscopy (ESGE) guideline. Endoscopy.

[21] Kakushima N., Kanemoto H., Tanaka M., Takizawa K., Ono H. (2014). Treatment for superficial non-ampullary duodenal epithelial tumors. World J. Gastroenterol..

[22] Sakon M., Takata M., Seki H., Hayashi K., Munakata Y. (2010). A novel combined laparoscopic-endoscopic cooperative approach for duodenal lesions. J. Laparoendosc. Adv. Surg. Tech..

[23] Singh K.L., Prabhu Gunjiganvi, Singh CAk, Moirangthem G.S. (2014). Isolated duodenal adenoma presenting as. J. Clin. Diagn. Res..

[24] Eisenberger C., Knoefel W., Peiper M., Yekebas E.F., Hosch S.B. (2004). Pancreas-sparing duodenectomy in duodenal pathology: indications and results. Hepato-Gastroenterology.

