# Supplementary material for: Case series of non-ampullary duodenal adenomas
Source: Ann Med Surg (Lond). 2021 Aug 20;69:102730. doi: 10.1016/j.amsu.2021.102730 (PMC8408424; doi:10.1016/j.amsu.2021.102730)
Supplement: Multimedia component 1 [file mmc1.docx]

| **PROCESS 2020 Checklist** | | | |
| --- | --- | --- | --- |
| **Topic** | **Item** | **Checklist Item Description** | **Page Number** |
| **Title** | **1** | CASE SERIES OF NON-AMPULLARY DUODENAL ADENOMAS | Title page |
| **Key Words** | **2** | - Sporadic Duodenal Adenoma - Familial Adenomatous Polyposis - Tubulo-Villous Adenoma | Page 2 |
| **Abstract** | **3a** | - Rare but important entity | Page 1 |
|  | **3b** | - Majority are treated with endoscopy, but they were operated due to large size |  |
|  | **3c** | Outcomes   - Offered one time treatment option |  |
|  | **3d** | Conclusion   - Passage of a small catheter from cystic duct stump helped in identification and sparing of ampulla in these large tumours |  |
| **Introduction** | **4** | - Adenomas are most found varieties in duodenum. They are uncommon and are found either sporadically or are associated with hereditary syndromes. - in the department of surgical gastroenterology and liver transplant at a tertiary care teaching hospital | Page 3 |
| **Methods** | **5a** | Registration   - State the research registry number in accordance with the Declaration of Helsinki - "Every research study involving human subjects must be registered in a publicly accessible database". This can be obtained from, for example, ResearchRegistry.com, ClinicalTrials.gov, or ISRCTN. - If a protocol already exists, state the corresponding registration number and access directions (e.g., website or journal, and include a hyperlink that is publicly accessible). It must be written in the English language. | researchregistry.com researchregistry 6837 |
|  | **5b** | Study Design   - Study is a case series. They are described as Case 1 and Case 2. - Cases were operated at single center and were non-consecutive. | Page 3-4 |
|  | **5c** | - The Sir Ganga Ram Hospital is a tertiary care teaching, research institution. - They data was collected from the data base of the hospital and it was a retrospectively collected data. | Page 3 |
|  | **5d** | Participants   - Two male patients were operated for duodenal adenomas, one was sporadic in nature and the other was associated with Peutz- Jeghers syndrome. - Methods used to ensure the de-identification of patient information. | Page 3 |
|  | **5e** | - Pre-Intervention Patient Optimization was done as both the patients were presented with obstructive symptoms | Page 3 |
|  | **5f** | Interventions   - Both the patients were treated surgically |  |
|  | **5g** | Intervention Details   - Patients were operated under general anesthesia, in supine position, and a ureteric catheter was used from cystic duct stump to identify and spare the ampulla in doing local resection of these large duodenal tumours. - Technique of Identification and sparing of ampulla by using passage of a catheter from cystic duct stump in large second part duodenal tumours is unique in this case series. |  |
|  | **5h** | Operator Details   - Both cases were operated by a team of experienced surgeons from the department of surgical gastroenterology and liver transplant. | Page 3 |
|  | **5i** | Quality Control   - Standard surgical technique was used in both the cases. |  |
|  | **5j** | Follow-Up   - The first case is in follow up for last 5 year and the second case for last 1 year. |  |
| **Results** | **6a** | Participants   - Two patients of benign duodenal tumours were operated for duodenal adenomas. |  |
|  | **6b** | Deviation from the Initial Management Plan   - No deviation was done |  |
|  | **6c** | Outcomes and Follow-Up   - Expected outcomes were attained and the reference literature was used to inform the expected outcomes. |  |
|  | **6d** | Intervention Adherence and Compliance   - None |  |
|  | **6e** | Complications and Adverse Events   - Precautionary measures including antibiotics and thromboprophylaxis were taken to prevent complications   . |  |
| **Discussion** | **7a** | - Summarized the key results. | Page 5-8 |
|  | **7b** | Relevant Literature and Placing the Results in Context   - Discussion of the relevant literature and similar published studies were done. | Page 8 |
|  | **7c** | Strengths   - Unique technique for identifying the ampulla   Weaknesses and Limitations   - Only case series of two cases |  |
| **Conclusions** | **8a** | Key Conclusions   - Surgery is a better and one-time option to treat large duodenal tumours than endoscopy |  |
|  | **8c** | Future Work   - Adding cholecystectomy and passage of a catheter from cystic duct across ampullary region helps in identification of ampulla and to avoid its injury during local resections of these large duodenal adenomas. |  |
| **Patient Perspective** | **9** | Patients were given the opportunity to share their perspective on the intervention, they received by a questionnaire. |  |
| **Informed Consent** | **10** | - The authors will provide evidence of consent, where applicable, and if requested by the journal. - Written informed consent was taken. |  |
| **Additional Information** | **11a** | - No conflicts of interest. |  |
|  | **11b** | - No sources of funding. |  |
|  | **11c** | Other Relevant Disclosures   - Institutional review board and ethical committee approval was not done according to the hospital policy on publishing case reports. - The cases were never presented at a conference or regional meeting. |  |
| **Clinical Images and Videos** | **12** | - Clinical images are available |  |
| **Referencing the Checklist** | **13** | - Include reference to the PROCESS 2020 publication by stating: 'This case series has been reported in line with the PROCESS Guideline' at the end of the introduction section (and include citation in the references section). | Page 3 |
